# 848. Discordant Chest Radiography and Computed Tomography Findings in Patients with Hematologic Malignancy and Invasive Mucormycosis: What Are the Prognostic Implications?

**DOI:** 10.1093/ofid/ofad500.893

**Published:** 2023-11-27

**Authors:** Sebastian Wurster, Alexander D Franklin, Dierdre B Axell-House, Ying Jiang, Dimitrios P Kontoyiannis

**Affiliations:** The University of Texas MD Anderson Cancer Center, Houston, Texas; Baylor College of Medicine, Houston, Texas; Houston Methodist Hospital, Houston, Texas; The University of Texas MD Anderson Cancer Center, Houston, Texas; The University of Texas MD Anderson Cancer Center, Houston, Texas

## Abstract

**Background:**

Invasive pulmonary mucormycosis (IPM) is a common and deadly mold infection in patients (pts) with hematological malignancies (HM). Comparing chest x-ray (CXR) to computed tomography (CT) in IPM offers the opportunity to evaluate the concordance of these two imaging studies and the clinical significance of discordant imaging.

**Methods:**

We reviewed all HM pts with CT-positive probable/proven IPM at MD Anderson Cancer Center (2000-2020) who had a concurrent CXR performed within 5 days of the reference CT. Concordant imaging was defined as a consolidation or mass-like lesion on CXR matching the suspicious CT lesion. In addition to survival curve analysis, a multivariate Cox's proportional hazard model was used to identify independent predictors of 84-day all-cause mortality after IPM symptom onset.

**Results:**

Among 44 included pts, 31 (70%) were neutropenic (< 500/µL). Only 24 pts (55%) had a matching lesion on CXR while the remaining 20 (45%) had discordant imaging. 20/29 pts (69%) with consolidation on CT and 9/12 pts (75%) with reverse halo sign or nodules with cavitary lesions had concordant CXR findings, whereas nodules without these features were rarely associated with concordant CXR findings (7/26, 27%). Although infection severity at baseline (APACHE II scores 15 vs. 16) was comparable, pts with concordant CXR lesions tended to have higher rates of intensive care unit admission during IPM therapy (67% vs. 45%, p = 0.15) and higher 84-day mortality (92% vs. 65%, p = 0.06) than those with discordant imaging. This difference was corroborated by survival curve analysis (Fig. 1A, p = 0.008), even when restricted to the 31 neutropenic pts (Fig. 1B, p = 0.049). APACHE II score > 18 at IPM diagnosis (adjusted hazard ratio [aHR] 3.09, p = 0.003), neutrophil recovery (aHR 0.29, p = 0.007), and presence of a concordant CXR lesion (aHR 2.61, p = 0.011) were independent predictors of 84-day mortality (Table 1).

**Figure 1**

**Figure:**
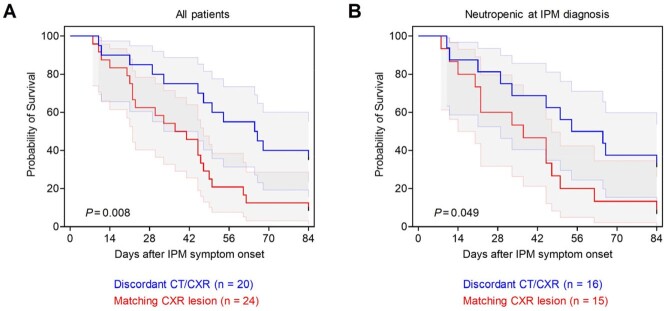

**Table 1**

**Figure:**
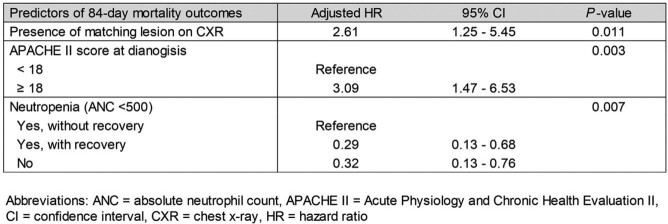

**Conclusion:**

Even when accounting for neutropenia status, a known confounder of radiographic imaging in IPM pts, visualization of IPM on CXR concordant to CT was an independent predictor of poor outcomes, possibly as a surrogate of extensive lesions and high fungal burden. If confirmed in larger cohorts, CXR might be used as a supportive prognostic staging tool in IPM pts, especially in resource-poor settings.

**Disclosures:**

**Dimitrios P. Kontoyiannis, MD, MS, ScD, PhD**, AbbVie: Board Member|Astellas: Grant/Research Support|Cidara: Board Member|Gilead: Grant/Research Support|Merck: Advisor/Consultant|Scynexis/MSGERC: Board Member

